# Intelligent Fault Diagnosis Method for Rotating Machinery Based on Recurrence Binary Plot and DSD-CNN

**DOI:** 10.3390/e26080675

**Published:** 2024-08-09

**Authors:** Yuxin Shi, Hongwei Wang, Wenlei Sun, Ruoyang Bai

**Affiliations:** School of Mechanical Engineering, Xinjiang University, Urumqi 830046, China; shiyuxinpayne@163.com (Y.S.); sunwenxj@163.com (W.S.); ruoyangbai@163.com (R.B.)

**Keywords:** fault diagnosis, rotating machinery, information entropy, recurrence binary plot

## Abstract

To tackle the issue of the traditional intelligent diagnostic algorithm’s insufficient utilization of correlation characteristics within the time series of fault signals and to meet the challenges of accuracy and computational complexity in rotating machinery fault diagnosis, a novel approach based on a recurrence binary plot (RBP) and a lightweight, deep, separable, dilated convolutional neural network (DSD-CNN) is proposed. Firstly, a recursive encoding method is used to convert the fault vibration signals of rotating machinery into two-dimensional texture images, extracting feature information from the internal structure of the fault signals as the input for the model. Subsequently, leveraging the excellent feature extraction capabilities of a lightweight convolutional neural network embedded with attention modules, the fault diagnosis of rotating machinery is carried out. The experimental results using different datasets demonstrate that the proposed model achieves excellent diagnostic accuracy and computational efficiency. Additionally, compared with other representative fault diagnosis methods, this model shows better anti-noise performance under different noise test data, and it provides a reliable and efficient reference solution for rotating machinery fault-classification tasks.

## 1. Introduction

Rolling bearings and gears constitute the crucial components of rotating machinery, and their health conditions significantly impact the performance, stability, and lifespan of mechanical equipment [1,2]. Vibration signals exhibit distinct variations across different fault types in rotating machinery. By implementing the real-time monitoring of vibration signals, it is possible to effectively prevent major accidents [3]. Hence, the development of a practical algorithmic tool to accurately and reliably diagnose the intricate, non-linear correlation between a raw vibration signal and the fault modes of rolling bearings and gears holds immense practical significance.

Traditional intelligent diagnostic algorithms based on expert systems are complex, time-consuming, and lack flexibility, rendering them inadequate for meeting the demands of industrial production in the era of internet big data. Exploring intelligent mechanical fault diagnosis methods can diminish the level of manual intervention and contribute to the safe and dependable operation of mechanical equipment [4]. In recent years, a frontier research direction called deep learning has proposed a novel approach for feature extraction based on neural networks. Compared with the classical shallow machine learning method, deep learning is a hierarchical representation learning method based on data. It learns deep abstract features by establishing neurons with multiple hidden layers, and it comprehensively considers the steps of feature extraction and classification so as to effectively improve the classification or prediction accuracy [5].

For the fault diagnosis tasks of mechanical equipment, a CNN aptly captures the intricate mapping relationship between various signals and corresponding fault states by learning from sensor-collected data [6]. Since the signal collected by the sensor is a one-dimensional time-domain signal, a large number of scholars have directly inputted the signal into a one-dimensional CNN to accomplish a fault diagnosis task. Wu et al. [7] proposed a method based on one-dimensional convolutional neural networks to autonomously learn valuable features from the original vibration signal of a rotor system, facilitating prompt and accurate fault detection. Mo et al. [8] integrated a learnable variational kernel into a one-dimensional CNN framework, with a focus on extracting crucial data features pertaining to faults, thereby achieving a commendable performance on small sample datasets. Junior et al. [9] introduced a multi-head, one-dimensional convolutional neural network that employed accelerometers measuring in two distinct directions to detect six different types of motor faults.

With the vigorous development of modern signal-processing technology, the method of preprocessing vibration signals at the front end has become widespread, yielding promising results. Deng et al. [10] employed an envelope demodulation analysis method for the feature extraction and fault diagnosis of a rolling bearing. Jin et al. [11] introduced a locomotive-bearing fault-diagnosis approach based on variational mode decomposition (VMD) and an improved convolutional neural network. Wang et al. [12] performed swarm decomposition (SWD) to decompose each segment of an original signal into several oscillation components (OC), and then they combined this with the improved MRDE algorithm to accomplish the intelligent identification and classification of various fault signals. Shao et al. [13] proposed an enhanced, modified, stacked autoencoder (MSAE) that employed adaptive Morlet wavelets for the automatic diagnosis of different fault types and severities in rotating machinery. Furthermore, Miao et al. [14] introduced a novel decomposition theory known as feature mode decomposition (FMD) for mechanical fault feature extraction.

Converting one-dimensional signals into feature images creates a link between low-level visual features and high-level visual features, which can emphasize recognizable characteristics. This technique is an efficient means of achieving information recognition and recovering high-dimensional features from low-dimensional information [15]. Wen et al. [16] employed a sliding window to normalize one-dimensional vibration signals into two-dimensional grayscale images which were then combined with a CNN, obviating the process of manual feature extraction. Xu et al. [17] input a time-frequency diagram, which was obtained through the continuous wavelet transform (CWT) of the vibration signal, into a CNN as a fault feature map for fault diagnosis. Shao et al. [18] retrained a CNN pretrained by ImageNet using a time-frequency diagram, effectively shortening the training time while extracting discernible fault features. Zhu et al. [19] transformed the vibration signals from multiple sensors into symmetrized dot pattern images (SDP) and employed a CNN to differentiate the rotor faults. Choudhary et al. [20] utilized a CNN to identify the thermal images of rolling bearings under five fault conditions, suggesting that infrared thermal imaging enables the non-contact early detection of faults without being influenced by speed. Zhi et al. [21] proposed a new entropy-aided meshing-order modulation (EMOM) indicator to capture the most sensitive modulation frequency area embedded in a signal, and they developed a wind-turbine fault-diagnosis method. Tang et al. [22] presented a composite model that combined improved mode decomposition, Gram angular summation field (GASF), and convolutional neural networks for the automated identification of the health statuses of complex mechanical systems. Xiong et al. [23] employed a mutual dimensionless theory and a similar Gram matrix to process bearing-fault vibration signals and subsequently integrated them with a convolutional neural network, which significantly reduced the training time. Bai et al. [24] proposed a frequency-domain Gram angular field (FDGAF) algorithm which could intelligently classify these feature images under the condition of 30 samples through a transfer learning network. Additionally, Bai [25] proposed a spectral Markov transition field (SMTF) algorithm which constructed the first-order Markov transition matrix of the frequency domain signal and represented the spectral characteristics of the vibration signal in image form.

In summary, although the above methods have achieved excellent results, the correlation characteristics between the time-series of fault signals have not been fully explored and utilized. Concurrently, there is still much room for improvement in the accuracy and computational efficiency of such a model. This paper proposes an intelligent fault diagnosis method for rotating machinery called RBP-DSD-CNN, which is based on a recurrence binary plot and a lightweight, deep, separable, dilated convolutional neural network. The main contributions of this paper are summarized as follows:

(a) A recursive quantization technique is introduced into the field of fault diagnosis. By leveraging the correlation characteristics of time-series data, feature information is extracted from the internal structures of fault signals, and this effectively enhances the expression capability of the features and mitigates the loss of weak information in the fault signals.
(b) Considering the characteristics of mechanical monitoring signals, a minimum mutual information method and the Cao method are employed to determine the optimal phase space-reconstruction parameters for each category.
(c) A DSD-CNN is developed for feature extraction and fault classification. This model adopts a lightweight structure, improving computational efficiency without compromising diagnostic accuracy.

## 2. Methodology

### 2.1. Recurrence Binary Plot

A recurrence plot (RP) is a recursive visualization method proposed by Eckmann [26] et al. on the basis of Poincare’s theorem, which is applied for the qualitative analysis of nonlinear dynamical systems. An RP portrays the recursive phenomenon of phase points and the evolution with time of the one-dimensional signals after the phase-space reconstruction in a two-dimensional image, which can intuitively characterize the dynamic characteristics implied by a system.

Time-series data can be classified using unique repetitive behaviors (e.g., periodicity and irregular periodicity). The essence of an RP is that it is a signal-processing method which is used to encode the cyclic behavior in a time-series into a two-dimensional image for representation. By applying the Takens embedding theorem, the phase-space reconstruction of the original time-series $\;\left\{x_{i}\;,i=1,2,\ldots ,N\right\}$ with a length of *N* is performed, and the phase-space vector obtained is as follows:

(1) $$Y{\;}_{i}=\left(x_{i},x_{i+\tau },\ldots ,x_{i+\left(m-1\right)\tau },\right)i=1,2,\ldots ,N-\left(m-1\right)\tau ,$$

where τ denotes the delay time and m represents the embedding dimension. The determination of τ and m will be described in detail in Section 2.2. On the basis of the reconstructed phase-space vector, the recursive matrix can be defined as follows:

(2) $$R_{ij}=\Theta \left(r-\left\|X_{i}-Y_{j}\right\|\right),$$

where Θ(•) is the Heavside function (as shown in Equation (3)), ‖•‖ represents the Euclidean norm, $\left\|X_{i}-Y_{j}\right\|$ is the distance between any two points in the reconstructed phase-space vector, and r is the distance threshold for determining whether there is a recursive phenomenon between two points in the phase-space. Obviously, the recursive matrix is a binary matrix consisting of 0 and 1.

(3) $$\Theta \left(x\right)=\{\begin{array}{c} 1,x\geq 0\\ 0,x<0 \end{array}.$$

When traversing any two points in the phase-space and after calculating all instances of $R_{ij}$, a recurrence binary plot (RBP) with black and white points symmetrically distributed around the main diagonal can be obtained.

**Figure 1:**
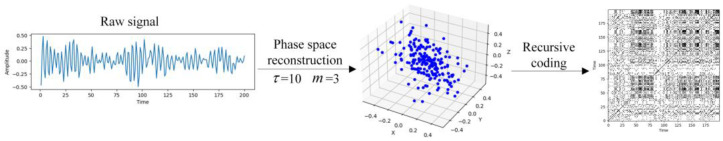
The flow chart of RBP.

**Figure 2:**
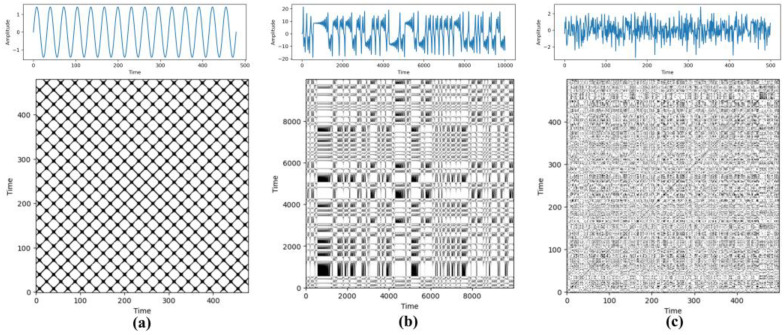
An RBP of diverse signals. (**a**) Sinusoidal signal. (**b**) X component of the Lorentz curve. (**c**) Gaussian white noise.

The flowchart illustrating the conversion of the raw signal to an RBP is shown in Figure 1. Figure 2 illustrates an RBP obtained through the recursive coding of diverse signals. With the increase in the non-stationarity, the detail texture becomes more significant.

### 2.2. Determination of the Phase-Space Reconstruction Parameters

The phase-space reconstruction serves as the foundation for a recursive quantitative analysis and recurrence plot construction. Therefore, determining the delay time τ and embedding dimension m is crucial. Takens posits that τ and m are theoretically independent. Thus, τ can be initially determined, upon which m is computed.

The minimum mutual information method was employed in this study to determine the delay time for two discrete information systems ($S={\left\{s_{i}\right\}}_{i=1}^{m}$ and $Q={\left\{q_{j}\right\}}_{j=1}^{n}$), and their mutual information is given by the following:

(4) I(S,Q)=H(S)+H(Q)−H(S,Q),

where H(S) and H(Q) represent the information entropies of the two systems defined by Shannon’s information theory and H(S,Q) denotes the joint probability distribution. When computing an actual time-series, S corresponds to the original time series x(n) and Q corresponds to the time-series x(n+τ) after being delayed by τ. When the mutual information value I(S,Q) reaches its first local minimum, it can be inferred that the corresponding τ value at this point is the optimal delay time, indicating that the time-series x(n) and x(n+τ) are maximally uncorrelated.

Subsequently, the Cao method [27] was utilized to estimate the embedding dimension, and this was an improvement to the False Nearest Neighbor (FNN) method, which was designed to reduce the influence of the manual threshold parameter selection. The definition of the Cao method is as follows:

(5) $$E\left(m\right)=\frac{1}{N-m\tau }\sum_{i=1}^{N-m\tau }a\left(i,m\right);E^{*}\left(m\right)=\frac{1}{N-m\tau }\sum_{i=1}^{N-m\tau }\left|x\left(i+m\tau \right)-x^{NN}\left(i+m\tau \right)\right|.$$

By calculating $E_{1}\left(m\right)=E\left(m+1\right)/E\left(m\right)$ under different values of m and introducing $E_{2}\left(m\right)=E^{*}\left(m+1\right)/E^{*}\left(m\right)$ as an auxiliary criterion, we could determine the optimal embedding dimension. If $E_{1}\left(m\right)$ and $E_{2}\left(m\right)$ are stabilized when m exceeds a certain value, the corresponding m value at this point can be considered the optimal embedding dimension.

**Figure 3:**
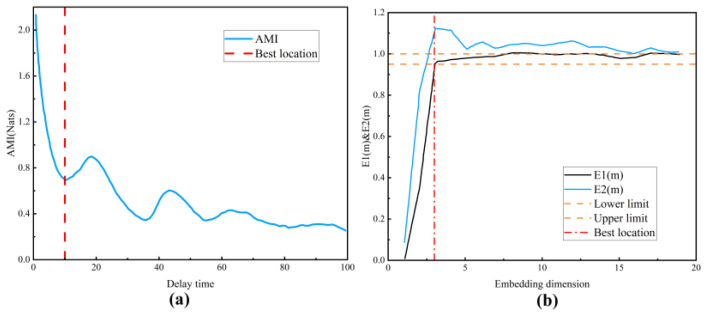
Determination of the delay time and embedding dimension. (**a**) Delay time. (**b**) Embedding dimension.

In Figure 3a, it is evident that the mutual information reaches its first minimum value when the delay time is 10, indicating the optimal delay time to be 10. Additionally, in Figure 3b, the stabilization of the changes in E1 and E2 occurs when the embedding dimension equals 3, suggesting the optimal embedding dimension to be 3.

### 2.3. Deep, Separable, Dilated Convolutional Neural Network (DSD-CNN)

Recently, deep learning methods have been widely used to unveil the hidden mapping between monitoring data and mechanical health status. However, these models are typically constructed for large-scale image classification tasks, and as the network depth increases, the computational and parameter complexity increase significantly. Considering the characteristics of mechanical monitoring signals, deep and separable convolutions (DSC) and dilated convolutions (DC) have been introduced into construct models.

A DSC [28] decomposes convolution operations into the following two steps: depth-wise convolution and point-wise convolution. Firstly, independent convolution kernels are applied to each channel of an input feature map to extract channel-specific features. Then, 1 × 1 convolution kernels are used for point-wise convolution, linearly combining and merging features from different channels to obtain the final output feature map, as follows:

(6) $$Conv{\left(W_{D},W_{P},x\right)}_{\left(i,j\right)}=\sum_{k}^{K}W_{k}\cdot x_{\left(i,j\right)}\cdot \left[W_{P},\sum_{m,n}^{M,N}W_{\left(m,n\right)}\cdot x_{\left(i+m,j+n\right)}\right],$$

where W represents the parameters of the kernel weight matrix. The input is denoted as x, while (i,j) signifies the coordination of output features. m,n,k, respectively, denote the width, height, and number of channels of the convolution kernel. The subscripts D and P represent depth-wise convolution and point-wise convolution operations, respectively.

A DC [29] expands a receptive field effectively and enhances a model’s performance by introducing gaps and holes within a convolutional kernel to augment the distance between the kernel and the input. The computational process of a DC is as follows;

(7) $$F_{i}=F_{i-1}+\left(k_{i}-1\right)\cdot r_{i}\cdot \prod_{n=1}^{i-1}s_{n},$$

where $F_{i}$ represents the receptive field of the convolution kernel in the *i*-th convolutional layer, $k_{i}$ denotes the size of the convolution kernel, $r_{i}$ is the dilation factor, and $s_{n}$ signifies the stride.

**Figure 4:**
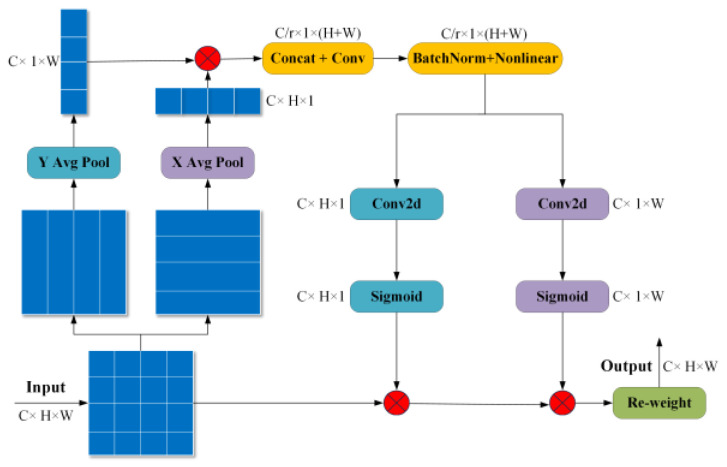
The structure of a CA.

A coordinate attention (CA) [30] is often regarded as a computing unit used to enhance the feature representation ability, and it can take any intermediate tensor as an input and obtain an output of the same size with enhanced representation ability. The structure of a CA is illustrated in Figure 4. In the first step, the coordinate information is embedded into the channel attention. The second step is to generate a coordinate attention.

**Figure 5:**
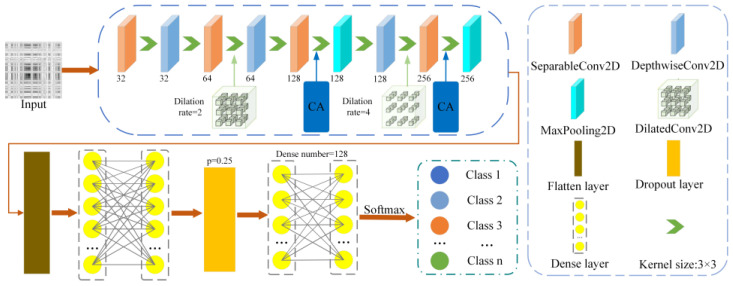
The structure of the DSD-CNN.

In this study, a lightweight convolutional neural network embedded with a CA was developed. The proposed model was designed to meet the requirements for both model performance and computational efficiency. The attention module enhanced the sensitivity of the feature location information, enabled the network to concentrate on learning essential features, and improved the training aggregation rate. The structure of the model is illustrated in Figure 5.

## 3. Overall Framework of the Proposed Approach

**Figure 6:**
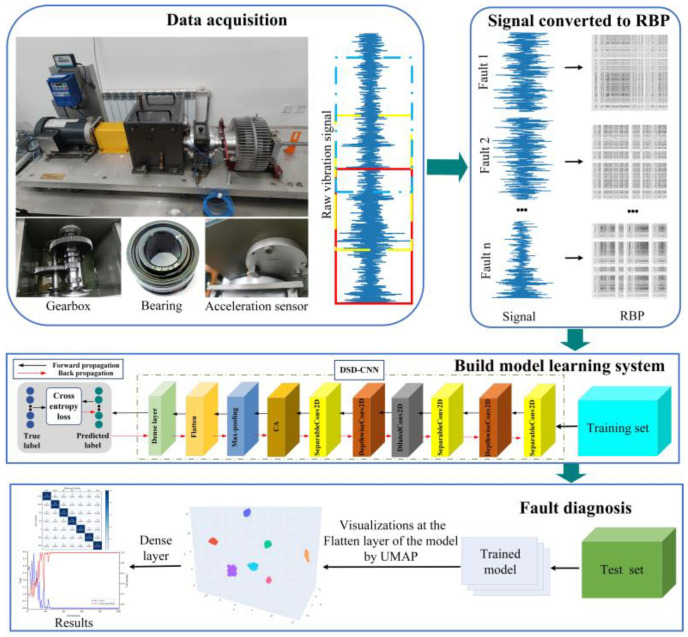
The proposed fault diagnosis framework.

Based on the above, a fault diagnosis framework was constructed as shown in Figure 6. The main steps were as follows.

Step 1: Data acquisition. The raw vibration signals of the different health states of the rolling bearings and gearboxes were collected from the test bench.

Step 2: Signal converted to an RBP. The raw vibration signal was divided into a series of one-dimensional samples through overlapping sampling, and then it was recursively encoded into an RBP as the input of the model.

Step 3: Build model learning system. The RBP, obtained in the previous step, was randomly divided into a training set and a test set at a ratio of 8:2, and afterwards, the model was trained with the training set.

Step 4: Fault diagnosis. The test set was input into the trained model to obtain the diagnosis results.

## 4. Experimental Study

In this study, experimental verification was conducted utilizing the bearing dataset of the Case Western Reserve University (CWRU) [31] and the gear dataset of the Wind Turbine Drivetrain Diagnostics Simulator (WTDS), which was collected in the authors’ laboratory.

### 4.1. Dataset Information

(1) CWRU: The data utilized in this study were sampled at a frequency of 12 KHz under the rotational speed of 1797 rpm from a drive-end bearing, and they contained normal (N), inner race fault (IF), outer race fault (OF), and ball fault (BF) types. The fault types could be further subdivided based on the fault sizes (14 mils and 21 mils). Consequently, seven different healthy states are considered. The specific information can be found in Table 1.

(2) WTDS: The data utilized in this study were collected via a three-axis acceleration sensor at a frequency of 20,480 Hz, which covered four fault types and contained a total of 2800 samples. The fault types could be further subdivided into seven health conditions, and the rotational speed was 2000 rpm. The detailed information is presented in Table 2.

**Figure 7:**
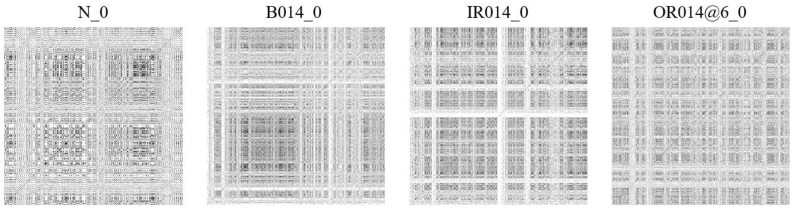
The RBP of the CWRU dataset.

**Figure 8:**
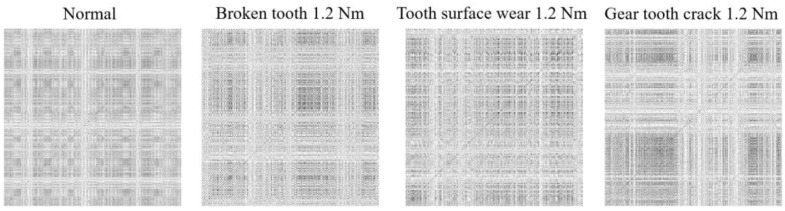
The RBP of the WTDS dataset.

The mechanical vibration signals collected above were reconstructed in phase space, as described of Section 2.1, and encoded into a recurrence binary plot. As depicted in Figure 7 and Figure 8, the corresponding health status is shown above each image.

### 4.2. Experimental Parameters

**Figure 9:**
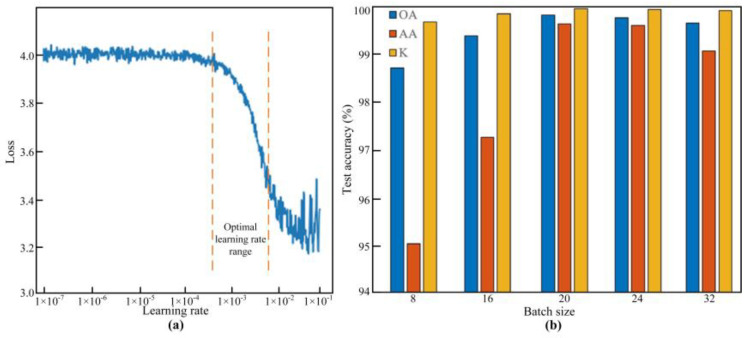
The determination of the model’s parameters. (**a**) Initial learning rate. (**b**) Batch size.

The model was built based on Pytorch-1.12.0 and trained by GPU. The operating system was Windows 11, with an Intel Core i5-13400F CPU running at 2.50 GHz. The GPU employed was an NVIDIA GeForce RTX 3060. For the selection of the initial learning rate, according to Leslie’s theory [32], we first set a smaller learning rate for the optimizer, and then we increased the learning rate in the data training of each batch until the loss began to become larger. The loss calculated by each batch was counted, and so the curves of the loss and learning rate are shown in Figure 9a. The optimal learning rate range could be obtained, as shown in the figure, and so the initial learning rate in this paper was set to 0.001 and the learning rate reduction strategy adopted “Reduce LR On Plateau”. In order to evaluate the influence of the different batch sizes on the classification performance of the model, overall accuracy (OA), average accuracy (AA), and a Kappa coefficient were introduced as quantitative evaluation indexes. The model was tested with batch sizes of 8, 16, 20, 24, and 32, respectively, and the results are shown in Figure 9b. When the batch size was 20, the OA, AA, and Kappa coefficient values were the highest. Therefore, the batch size was set to 20. While the model’s weight-update back-propagation algorithm was small batch gradient descent, the loss function was the cross-entropy loss function, and the optimizer was Adam.

### 4.3. Model Rationality Analysis

**Figure 10:**
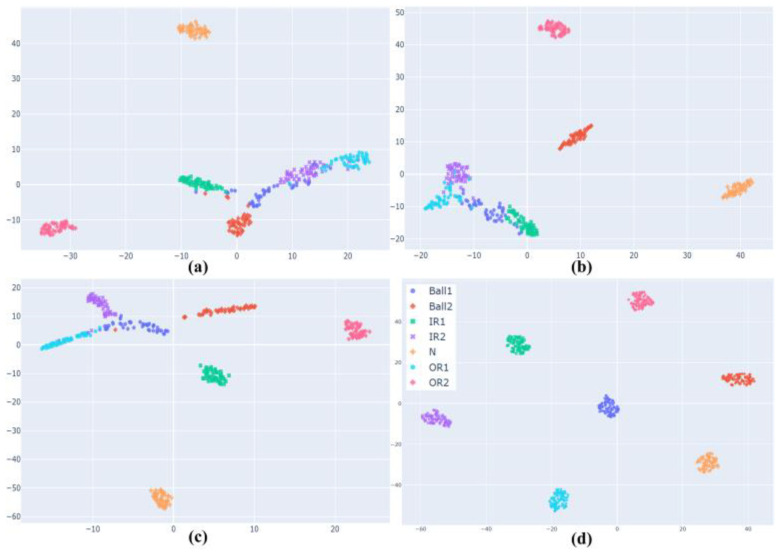
A visualization of the results of the ablation experiment. (**a**) Removal of the SeparableConv block. (**b**) Removal of the DepthwiseConv block. (**c**) Removal of the CA module. (**d**) The DSD-CNN.

The ablation experiment was carried out by gradually removing some components in the DSD-CNN to observe their effects on the performance of the model so as to evaluate the importance of these components and explain the rationality of the model’s settings. The dataset shown in Table 1 was selected as the model’s input, and the experimental results are shown in Figure 10 and Table 3, respectively.

It can be seen from Table 3 that the SeparableConv block had the greatest influence on the performance and computational efficiency of the model. In Figure 10a, after removing this component, the model could only clearly classify the normal and 21 mils outer race faults, and the remaining health state boundaries were blurred and difficult to distinguish. Compared with the SeparableConv block, the DepthwiseConv block and the CA module had less impact on the performance of the model, and especially after removing the CA module, the model could still achieve a classification accuracy of over 96%. However, considering the influence of computational efficiency, the existence of the above two components was necessary. In general, the proposed DSD-CNN was reasonable and could balance computational efficiency and classification performance.

### 4.4. Comparison Method

To verify the effectiveness of the proposed method, the diagnostic performance and computational efficiency of the proposed model were compared with other algorithms, including classical deep convolutional neural networks and representative lightweight models.

(1) ResNet [33]: In this study, the widely used ResNet50 was selected as a comparison method, and it consisted of 4 conv blocks, 12 identity blocks, a total of 49 convolutional layers, and 1 Softmax classifier.
(2) ShuffleNetV2 [34]: ShufflenetV2 with a 2.0× output channel was constructed for comparison, and it consisted of 17 convolution layers and 2 pooling layers with the original convolution kernel size of 112 × 112, and it was halved to 7 × 7 layer by layer.
(3) EfficientNet [35]: Considering the model’s depth and computational efficiency, EfficientNetB3 was selected as a comparison method, and it was mainly composed of two convolutional layers and seven stacked MBConv layers.
(4) DenseNet [36]: The DenseNet 161 used in this study mainly consisted of four dense blocks and three transition layers.
(5) Light-weight CNN (LW-CNN) [37]: The LW-CNN comprised a convolution layer consisting of eight filters of 3 × 3 size, followed by a maxpool layer and a fully connected layer with a softmax activation.

Moreover, all of the above methods shared the same experimental parameters as the proposed methods.

### 4.5. Case Study I

In this section, the gear-fault data collected from the WTDS test rig were employed to validate the model. The maximum number of iterations was set to 100.

#### 4.5.1. Model Performance Analysis

**Figure 11:**
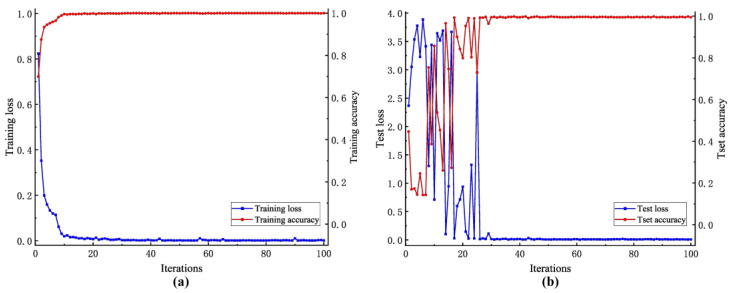
Loss and accuracy curves of the DSD-CNN based on the WTDS dataset. (**a**) Training set. (**b**) Test set.

The model loss and accuracy curves for the WTDS dataset are shown in Figure 11. It could be concluded that the DSD-CNN proposed in this paper demonstrated the characteristics of rapid model aggregation and high diagnostic accuracy. For the training set, after nine iterations, the training accuracy reached 99.56%. Subsequently, the training accuracy reached 100% after 32 iterations, indicating that the model had converged on the training set. For the test set, after approximately 26 iterations, the model had achieved a 99% test accuracy. Furthermore, after 38 iterations, the test accuracy reached 99.82%, indicating that the proposed model had been trained to the optimal level and had good classification ability for gear-fault signals.

For the purpose of evaluating the performance of the proposed model, indicators such as precision, recall, and F1-score were introduced in the study. The classification results of the model on the WTDS dataset are presented in Table 4.

**Figure 12:**
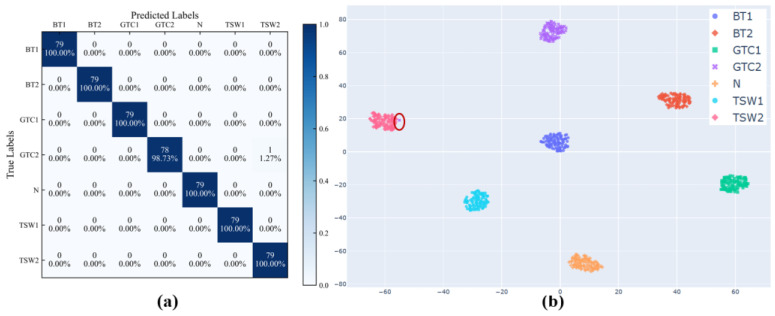
Classification results of the WTDS dataset. (**a**) Confusion matrix. (**b**) Dimensionality reduction visualization by the T-SNE.

The corresponding confusion matrix is depicted in Figure 12a, and the results demonstrated that the model could accurately classify fault states. T-distributed Stochastic Neighbor Embedding (T-SNE) [38] is a nonlinear, unsupervised dimensionality reduction method which could reduce the vector dimension and capture the complex manifold structure of the original data. By means of the T-SNE, the output features at the dense layer of the model were visualized in two-dimensional space, as shown in Figure 12b. Despite the fact that there were some errors in reducing the high-dimensional feature information to two-dimensional space, the purpose of the two-dimensional visualization was to provide an intuitive means for judging whether the features extracted by the model were discriminative. As presented in Figure 10b, the model misclassified one sample in the 1.2 Nm gear-tooth crack as 1.2 Nm tooth-surface wear (as shown in the red ellipse annotation in Figure 12b). A possible reason is that the two fault categories were distributed on the gear surface. When the gear was engaged, there was a gap in the meshing line, and the collected vibration signals were similar; however, these were different from the vibration impact generated when the broken tooth was engaged, resulting in a misclassification by the model.

**Figure 13:**
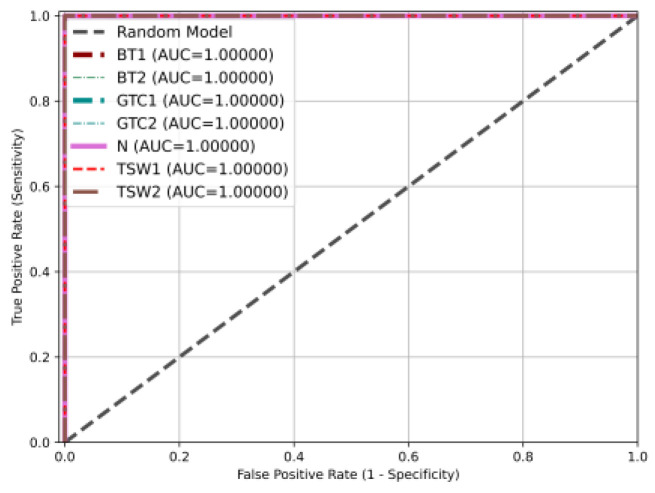
The ROC curves of the DSD-CNN based on the WTDS dataset.

An ROC curve refers to a receiver operating characteristic curve, which comprehensively reflects the sensitivity and specificity of continuous variables. A curve was drawn with 1-specificity as the abscissa and sensitivity as the ordinate. The larger the area under the curve, namely, the larger the AUC value, the higher the accuracy of the experimental results. The results demonstrated that the AUC value of the model for each type of fault on the WTDS test rig was 1, as shown in Figure 13.

Through a comprehensive analysis, it could be concluded that the network proposed in this paper possessed a strong feature extraction ability and could focus on the essential information contained in an input feature map. Simultaneously, the inter-class separation and intra-class compactness were achieved, which indicated the superiority and feasibility of the proposed model for the fault diagnosis of rotating machinery.

#### 4.5.2. Comparison between Different Methods

**Figure 14:**
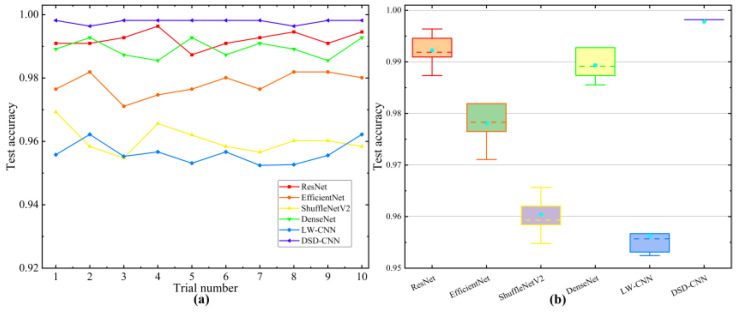
Comparison of the different methods on the WTDS test rig. (**a**) Test accuracy curve. (**b**) Boxplots.

Each model experiment was repeated 10 times to reduce the effect of randomness. The comparison experiment results are depicted in Figure 14, and the average test accuracy of the experiment is represented in Table 5.

In a comparative experiment conducted on the WTDS test rig, all six models exhibited good diagnostic accuracy and achieved test accuracies of over 95%. Compared with the deep models ResNet and DenseNet, the proposed method achieved better diagnostic accuracy than the above models while saving on computational efficiency. In addition, compared with the lightweight models ShuffleNet and LW-CNN, the proposed method achieved better results at the expense of some computational efficiency. In general, the proposed method achieved a higher classification accuracy and faster training speed through using a lightweight convolutional neural network embedded with an attention module compared with the above classic deep algorithm and representative lightweight network.

#### 4.5.3. Anti-Noise Analysis

In practical industrial production, mechanical equipment usually runs under variable working conditions, and the data collected from the equipment contain certain background noise interference. In order to further study the performance of the algorithm in a noisy environment, different levels of Gaussian white noise were added to the original test dataset, and the signal-to-noise ratios (SNRs) were 8 dB and 4 dB, respectively. The noisy test data were input into the above well-trained model for classification. Table 6 shows the classification results of the model under different noise levels.

It can be seen from the results that the diagnostic accuracy of all methods was greatly reduced compared with that before the noise addition. This was because the discriminant information in the original vibration signal would be disturbed and covered by noise, resulting in a large difference in the distribution of the training data and test data, thus further increasing the difficulty of the model in learning the discriminant features. When the signal-to-noise ratio was high (SNR = 8 dB), all methods obtained an average accuracy higher than 87%, showing a certain anti-noise ability and generalization performance. The proposed method achieved an average diagnostic accuracy of more than 95%, indicating that the proposed model could maintain good feature learning ability under small noise interference.

### 4.6. Case Study II

In recent years, the CWRU dataset has been widely adopted by numerous researchers in fault diagnosis experiments to validate the performance of the studied models. The experimental conditions remained the same as those of the WTDS experiment described above.

#### 4.6.1. Model Performance Analysis

**Figure 15:**
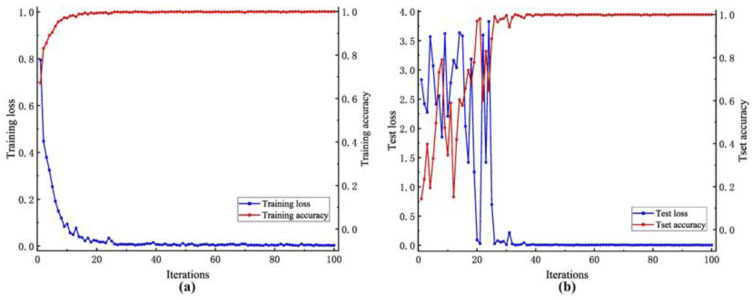
Loss and accuracy curves of the DSD-CNN based on the CWRU dataset. (**a**) Training set. (**b**) Test set.

The loss and accuracy curves of the model based on the CWRU dataset are shown in Figure 15. For the training set, after approximately 18 iterations, the model had achieved a 99.5% training accuracy. Furthermore, after 33 iterations, the training accuracy reached 100%. For the test set, after 26 iterations, the test accuracy reached 99%. This further improved to 100% after approximately 33 iterations, marking the optimal level of model training. Precision, recall and F1-score were still employed to evaluate the performance of the model. The results are represented in Table 7.

**Figure 16:**
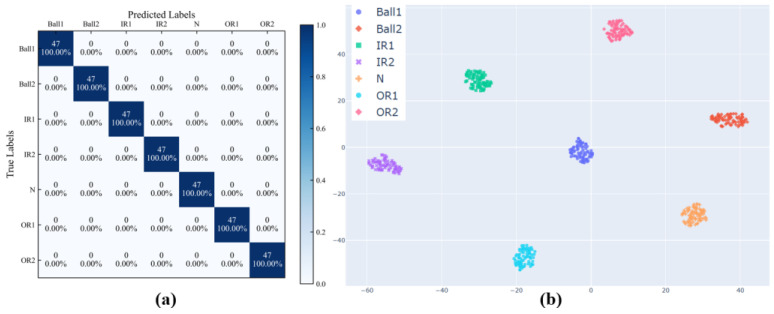
Classification results of the CWRU dataset. (**a**) Confusion matrix. (**b**) Dimensionality reduction visualization by the T-SNE.

The classification results of the model are depicted in Figure 16. The results exhibited that the model could accurately classify each fault state, with the value of the three indicators for each fault type being 100%.

#### 4.6.2. Comparison between the Different Methods

**Figure 17:**
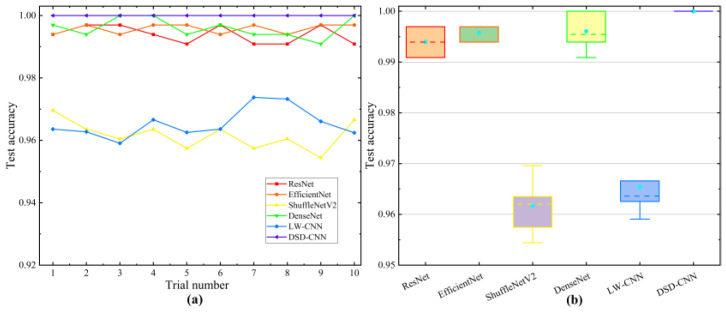
Comparison of the different methods on the CWRU test rig. (**a**) Test accuracy curve. (**b**) Boxplots.

Ten comparative experiments were carried out. The comparison test results are shown in Figure 17, and the average test accuracy is presented in Table 8.

In the above comparative experiments, the diagnostic accuracy was higher than 91%. Compared with the other approaches, the model proposed in this paper demonstrated a superior performance, achieving a diagnostic accuracy of 100%. Concurrently, the computational efficiency was improved under the premise of ensuring the performance of the model.

#### 4.6.3. Anti-Noise Analysis

After the model was well-trained on the training dataset, two different Gaussian white noises were also added to the test data, and the anti-noise performance of the different methods was studied. The diagnostic results are shown in Table 9.

The classification accuracy of all algorithms decreased with the increase in noise level. The anti-noise performance of the model trained by the CWRU dataset was significantly weaker than that of the WTDS dataset. The reason may have been that the gear to be diagnosed in the WTDS test rig was connected to the motor through another pair of gears, and additional modulation components were introduced in the transmission path. Therefore, the data distribution was quite different, which was equivalent to improving the anti-noise performance of the model.

## 5. Conclusions

In this paper, an intelligent diagnosis method based on a recurrence binary plot and a DSD-CNN is proposed for the condition-monitoring of mechanical equipment. In order to better extract the data structure of the mechanical monitoring signal, a recursive quantization technique was used to encode the vibration signal, and optimal phase-space reconstruction parameters were determined. A lightweight convolutional neural network was developed for feature extraction and fault classification. Through the comparative analysis of two experimental cases, the superiority and robustness of the proposed method were verified. Overall, the experimental results show that the proposed method significantly improves the accuracy and computational efficiency of mechanical fault classification. In addition, the proposed method shows better anti-noise performance under different noise test data compared with other approaches.

In practical engineering scenarios, acquiring sufficient labeled data for model training often proves infeasible. Therefore, the research work of domain adaptation algorithms based on transfer-learning for variable working conditions and cross-machines has been started.

## Figures and Tables

**Table 1:** Details of the experimental dataset selected from the CWRU data.

Label	Fault Information	Speed	Length of Sample	Size of Sample	Index
Ball1	B014_0	1797 rpm	1000	240	0
Ball2	B021_0		1000	240	1
IR1	IR014_0		1000	240	2
IR2	IR021_0		1000	240	3
N	N_0		1000	240	4
OR1	OR014@6_0		1000	240	5
OR2	OR021@6_0		1000	240	6

**Table 2:** Details of the experimental dataset selected from the WTDS data.

Label	Fault Information	Load	Length of Sample	Size of Sample	Index
BT1	Broken tooth	0.4 Nm	1200	400	0
BT2	Broken tooth	1.2 Nm	1200	400	1
GTC1	Gear tooth crack	0.4 Nm	1200	400	2
GTC2	Gear tooth crack	1.2 Nm	1200	400	3
N	Normal	0 Nm	1200	400	4
TSW1	Tooth surface wear	0.4 Nm	1200	400	5
TSW2	Tooth surface wear	1.2 Nm	1200	400	6

**Table 3:** The results of the ablation experiment.

Type	Accuracy	Precision	Recall	F1-Score	Total Parameters	Total FLOPs
Removal of the SeparableConv block	73.25%	73.31%	73.25%	73.25%	5,716,980	631.29 MFLOAPs
Removal of the DepthwiseConv block	87.84%	89.65%	87.84%	87.84%	6,517,728	719.71 MFLOAPs
Removal of the CA module	96.35%	96.61%	96.35%	96.35%	6,467,472	714.15 MFLOAPs
**DSD-CNN**	**100%**	**100%**	**100%**	**100%**	**6,558,192**	**724.18 MFLOAPs**

**Table 4:** The classification results of the DSD-CNN on the WTDS test rig (%).

Label	BT1	BT2	GTC1	GTC2	N	TSW1	TSW2	Average
Precision	100.00	100.00	100.00	100.00	100.00	100.00	98.75	99.82
Recall	100.00	100.00	100.00	98.73	100.00	100.00	100.00	99.82
F1-score	100.00	100.00	100.00	99.36	100.00	100.00	100.00	99.82
Support	79	79	79	79	79	79	79	553

**Table 5:** Accuracy comparison of the different classification methods on the WTDS test rig.

Method	Accuracy (%)	Total Parameters	Total Flops
ResNet	98.22 ± 0.24	23,528,522	4.12 GFLOAPs
EfficientNet	97.81 ± 0.37	10,706,991	987.18 MFLOAPs
ShuffleNetV2	96.04 ± 0.41	5,359,339	591.8 MFLOAPs
DenseNet	98.93 ± 0.29	28,681,000	7.82 GFLOAPs
LW-CNN	95.63 ± 0.33	2,397,087	363.59 MFLOAPs
**DSD-CNN**	**99.78 ± 0.07**	**6,558,192**	**724.18 MFLOAPs**

**Table 6:** Anti-noise analysis of the different methods using the WTDS dataset (%).

Method	Average Accuracy (4 dB)	Average Accuracy (8 dB)
ResNet	79.44 ± 3.12	92.79 ± 1.89
EfficientNet	72.29 ± 5.26	91.71 ± 3.22
ShuffleNetV2	77.89 ± 5.08	90.87 ± 4.23
DenseNet	85.14 ± 3.87	94.31 ± 1.83
LW-CNN	75.67 ± 3.79	91.29 ± 2.61
**DSD-CNN**	**87.82 ± 2.84**	**95.17 ± 1.14**

**Table 7:** The classification results of the DSD-CNN on the CWRU test rig (%).

Label	Ball1	Ball2	IR1	IR2	N	OR1	OR2	Average
Precision	100.00	100.00	100.00	100.00	100.00	100.00	100.00	100.00
Recall	100.00	100.00	100.00	100.00	100.00	100.00	100.00	100.00
F1-score	100.00	100.00	100.00	100.00	100.00	100.00	100.00	100.00
Support	47	47	47	47	47	47	47	329

**Table 8:** Accuracy comparison of the different classification methods on the CWRU test rig.

Method	Accuracy (%)	Total Parameters	Total Flops
ResNet	99.39 ± 0.29	23,528,522	4.12 GFLOAPs
EfficientNet	99.57 ± 0.16	10,706,991	987.18 MFLOAPs
ShuffleNetV2	96.17 ± 0.46	5,359,339	591.8 MFLOAPs
DenseNet	99.61 ± 0.32	28,681,000	7.82 GFLOAPs
LW-CNN	96.54 ± 0.45	2,397,087	363.59 MFLOAPs
**DSD-CNN**	**100.00 ± 0.00**	**6,558,192**	**724.18 MFLOAPs**

**Table 9:** Anti-noise analysis of the different methods on the WTDS dataset (%).

Method	Average Accuracy (4 dB)	Average Accuracy (8 dB)
ResNet	73.43 ± 2.45	91.89 ± 2.82
EfficientNet	70.92 ± 4.77	92.25 ± 1.93
ShuffleNetV2	74.27 ± 3.39	90.57 ± 4.21
DenseNet	86.58 ± 3.15	95.01 ± 2.18
LW-CNN	74.43 ± 4.25	91.12 ± 2.64
**DSD-CNN**	**86.97 ± 2.64**	**95.45 ± 1.72**

## Data Availability

The public dataset from CWRU can be found at https://engineering.case.edu/bearingdatacenter (accessed on 1 March 2022). The signals collected from the WTDS test rig in the authors’ laboratory are owned by the authors’ institution, and they are not allowed to be used in a public way.
